# The Randomized, Multicenter, Open-Label, Controlled POLBOS 3 Trial Comparing Regular Drug-Eluting Stents and the Sirolimus-Eluting BiOSS LIM C Dedicated Coronary Bifurcation Stent: Four-Year Results

**DOI:** 10.3390/biomedicines12050938

**Published:** 2024-04-23

**Authors:** Robert J. Gil, Adam Kern, Krystian Bojko, Aneta Gziut-Rudkowska, Dobrin Vassilev, Jacek Bil

**Affiliations:** 1 Department of Cardiology, State Medical Institute of the Ministry of Interior and Administration, 02-507 Warsaw, Poland; robert.gil@cskmswia.gov.pl (R.J.G.); aneta.gziut@cskmswia.gov.pl (A.G.-R.); 2 Department of Cardiology and Internal Medicine, School of Medicine, Collegium Medicum, University of Warmia and Mazury, 10-719 Olsztyn, Poland; adam.kern@uwm.edu.pl (A.K.); krystian.bojko@uwm.edu.pl (K.B.); 3 Medica Cor University Hospital, University of Ruse, 7017 Ruse, Bulgaria; dobrinv@gmail.com; 4 Department of Invasive Cardiology, Centre of Postgraduate Medical Education, 01-826 Warsaw, Poland

**Keywords:** non-LM bifurcation, percutaneous coronary intervention, high-risk PCI, long-term data

## Abstract

This multicenter, randomized study aimed to compare the sirolimus-eluting BiOSS LIM C dedicated coronary bifurcation stent with second-generation -limus drug-eluting stents (rDESs) in the treatment of non-left main (non-LM) coronary bifurcation. The deployment of a single stent in the main vessel–main branch across a side branch was the default strategy in all patients. The primary endpoint was the rate of major cardiovascular events (cardiac death, myocardial infarction, and target lesion revascularization) at 48 months. We enrolled 230 patients, allocating 116 patients to the BiOSS LIM C group and 114 patients to the rDES group. Most procedures were elective (BiOSS vs. rDES: 48.3% vs. 59.6%, *p* = 0.09) and performed in bifurcations within the left anterior descending/diagonal branch (BiOSS vs. rDES: 51.7% vs. 61.4%, *p* = 0.15). At 48 months, there were no statistically significant differences between the BiOSS and rDES groups in terms of major adverse cardiovascular events (MACE), cardiac death, myocardial infarction (MI), or target lesion revascularization (TLR) as follows: MACEs—18.1% vs. 14.9%, HR 1.36, 95% CI 0.62–2.22, and *p* = 0.33; cardiac death—4.3% vs. 3.5%, HR 1.23, 95% CI 0.33–4.56, and *p* = 0.75; MI—2.6% vs. 3.5%, HR 0.73, 95% CI 0.17–3.23, and *p* = 0.68; and TLR—11.2% vs. 7.9%, HR 1.66, 95% CI 0.75–3.71, and *p* = 0.21. The implantation success rate of the BiOSS LIM C stent was very high, and the cumulative MACE rates were promising. The POLBOS 3 trial sets an important benchmark for treating non-LM coronary bifurcations (ClinicalTrials.gov NCT03548272).

## 1. Introduction

Advancements in various technologies employed in percutaneous coronary intervention (PCI) have prompted greater efforts to address increasingly complex coronary lesions, encompassing even intricate coronary bifurcations. Among the most demanding types of complex lesions, coronary bifurcations present significant challenges. It is noted that complex lesions, including bifurcations, are linked to poorer immediate and long-term results compared to simpler lesions [1,2,3].

The heightened risk associated with coronary bifurcation lesions has sparked ongoing discussion regarding their management; when is it imperative to address the side branch (SB) alongside the main vessel (MV)? Two approaches are prominent: the provisional stent placement strategy, in which the focus is on treating the MV initially and intervening in the SB only if it faces imminent closure or closure occurs, and the dedicated two-stent strategy which, conversely, entails planning for the simultaneous treatment of both the MV and SB from the very beginning [4,5,6].

All things considered, there still might be a place for dedicated bifurcation stents. One such stent is the BiOSS (Bifurcation Optimization Stent System, Balton, Poland), which has been investigated for over 15 years. The initial BiOSS stent was bare metal (stainless steel), but it was soon followed by the introduction of a paclitaxel-eluting variant known as the BiOSS Expert stent (CE Mark 2010). Subsequently, in 2012, the sirolimus-eluting BiOSS LIM stent was developed. Results obtained from registries, clinical randomized trials such as POLBOS 1 and POLBOS 2, as well as everyday clinical practice were deemed satisfactory. However, room for improvement remained [7,8,9]. Relatively large neointimal growths associated with thick struts (120 μm) were a reason to design a cobalt–chromium sirolimus-eluting version—the BiOSS LIM C stent [10].

After both successful preclinical testing and a first-in-human study of the BiOSS LIM C stent, a randomized study (POLBOS 3) was designed [11,12,13].

This study aimed to compare the BiOSS LIM C with - second-generation -limus drug-eluting stents (regular DESs and rDESs) in the treatment of non-left main (non-LM) coronary bifurcation. This study was designed to include five hundred eighteen patients with symptomatic stable coronary artery disease or NSTE-ACS who qualified for PCI in non-LM coronary bifurcation [14]. However, due to the outbreak of the COVID-19 pandemic and an extremely low enrolment rate, the decision was made to stop the study at 230 patients. Here, we present our 4-year clinical results.

## 2. Materials and Methods

### 2.1. Device

**Figure 1:**
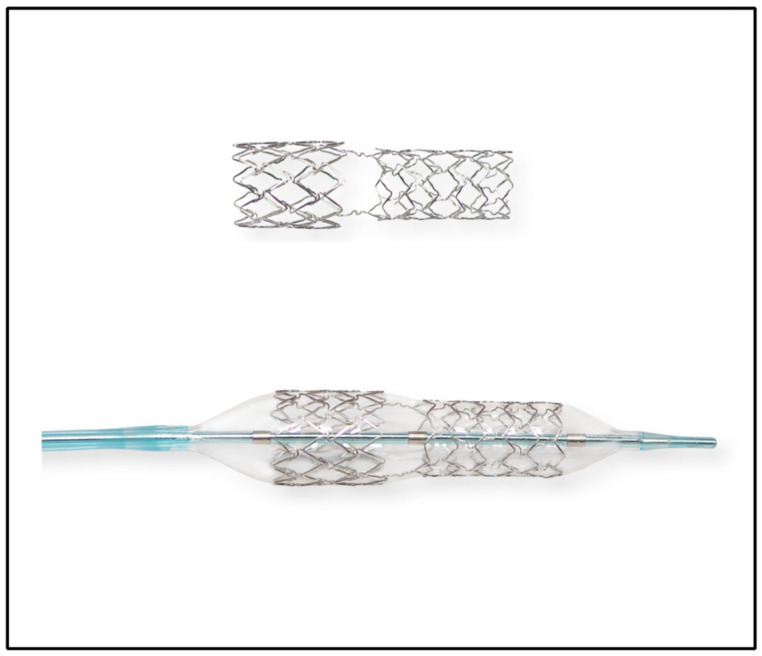
BiOSS LIM C structure.

The BiOSS LIM C stent is a dedicated bifurcation stent crafted from a cobalt–chromium alloy with a strut thickness of 70 µm. It releases sirolimus (1.3 µg/mm^2^) from a biodegradable coating in a process lasting around 8 weeks. This stent comprises two distinct parts with varying diameters; it is wider proximally and smaller distally. The proximal segment is always shorter than the distal one (45% vs. 55%, respectively). The ratio of the diameters of the proximal part (ranging from 3.0 to 5.0 mm) and the distal part (ranging from 2.5 to 4.0 mm) varies between 1.15 and 1.3, ensuring physiological compatibility and optimal flow dynamics. A middle zone of 2.0 to 2.4 mm accommodates two connecting struts to facilitate an opening for the SB. BiOSS stents are available in three lengths: 16, 19, and 24 mm (Figure 1) [11].

### 2.2. Study Population and Study Design

This was a multicenter (two centers in Poland) randomized, controlled study that enrolled patients between 2018 and 2020. Patients were enrolled if an informed consent form was signed and all inclusion criteria and no exclusion criteria were met. The inclusion and exclusion criteria are provided in Table 1 [14].

Patients were randomized 1:1 to a BiOSS LIM C stent group versus a regular DES (rDES) group. The following rDESs were used: Xience (Abbott Laboratories, Warsaw, Poland), Resolute Onyx (Medtronic Poland, Warsaw, Poland), Orsiro (Biotronik Polska, Poznan, Poland), Synergy (Boston Scientific Polska, Warsaw, Poland), and Promus Elite (Boston Scientific). This study’s protocol was compliant with SPIRIT guidelines [15]. An independent Ethics Committee approved the study protocol (No. 14/2018) (ClinicalTrials.gov NCT03548272).

### 2.3. Study Methodology

The study procedure was described in detail earlier [14]. In short, single-stent deployment in the MV-MB across the SB was the default strategy (provisional T-stenting, PTS) in all patients. We defined the MB as the part of the MV below the emergence of the SB. Bifurcation lesions were assessed according to the Medina classification, using an index of 1 for stenosis greater than 50% and 0 for no stenosis (as per a visual estimation) [16]. There was no restriction regarding lesion length in patient selection. If required, an additional stent could be implanted. A stent in an SB was allowed only if there was proximal residual stenosis greater than 70% after balloon dilatation and/or significant flow impairment after MV—MV stenting and/or a flow-limiting dissection.

The implantation protocol for bifurcation was as follows:

1. Wiring the MB and SB;
2. MV-MB predilatation according to the operator’s decision (with a balloon–MB diameter ratio close to 1:1);
3. SB predilatation according to the operator’s decision (only with a small balloon);
4. Stent implantation (with inflation for at least 20 s);
5. Use of the proximal optimization technique (POT);
6. SB postdilatation or SB stent implantation if required;
7. A final kissing balloon inflation at the operator’s discretion (mandatory only if the two-stent technique was applied);
8. A final use of the proximal optimization technique (re-POT).

For patients diagnosed with acute coronary syndrome (ACS), a loading dose of ticagrelor (180 mg) or clopidogrel (600 mg) was administered, with the option of an loading dose of additional acetylsalicylic acid (ASA) if necessary (300 mg). Prior to the planned procedures, all patients received ASA (75 mg/24 h) and clopidogrel (75 mg/24 h) starting seventy-two hours before percutaneous coronary intervention (PCI). PCI procedures were conducted using standard protocols via either radial or femoral access, utilizing 6 or 7 Fr guiding catheters. Following arterial sheath insertion, each patient received unfractionated heparin (100 IU/kg), with additional boluses administered to maintain an activated clotting time > 250 s. Dual antiplatelet therapy (75 mg q.d. ASA and 75 mg q.d. of clopidogrel or 90 mg b.i.d. of ticagrelor) was prescribed for 12 months.

Patients underwent evaluations of their troponin I (TnI), creatine kinase (CK), and CK-MB levels before the procedure and at 6 and 24 h post procedure. An assessment of periprocedural myocardial infarction (type 4a) was conducted following the criteria outlined in the fourth universal definition, with patients diagnosed with non-ST-segment-elevation myocardial infarction (NSTEMI) excluded from this evaluation [17].

### 2.4. Follow-Up

Follow-up visits were performed at 30 days, 6 months, 12 months, 24 months, 36 months, and 48 months. At the clinical follow-up visit, clinical status (including angina symptoms according to the Canadian Cardiovascular Society classification), adverse events according to the protocol, hospitalization, and medication intake were evaluated.

### 2.5. Endpoints

The primary endpoint was the rate of major cardiovascular events (cardiac death, myocardial infarction, and target lesion revascularization) at 48 months. The secondary endpoints were as follows: all-cause death; cardiovascular death—all deaths considered cardiovascular death if not proved otherwise; myocardial infarction; target lesion revascularization; and stent thrombosis at 12 and 48 months.

An independent Clinical Event Committee was created by three cardiologists who were otherwise not involved in the study. Each clinical event was adjudicated independently and blindly by two members of the Committee. In case of a disagreement, the third member was involved, and a joint agreement was reached.

### 2.6. Statistics

The original sample size calculation was as follows: assuming a noninferiority margin (delta) of 8%, a total sample size of 518 patients (with a drop-out rate of 5%), 259 patients per group, were calculated to be necessary (type I error: 0.05; type II error: 0.2; statistical power: 80%).

Descriptive statistics are shown as mean values with standard deviation values, minimum values, median values with an interquartile range, and maximum values for continuous variables; categorical variables are presented as counts and percentages. Pearson’s Chi-squared test or Fisher’s exact test were used to compare categorical variables between the 2 subgroups (BiOSS and rDES patients). We applied Fisher’s exact test if at least one of the subgroups had a count = 0. Wilcoxon’s rank-sum test was performed to compare continuous variables between the 2 subgroups (BiOSS and rDES patients). A *p*-value < 0.05 was judged statistically significant.

Kaplan–Meier estimators with 95% confidence intervals (CIs) were used to compare 48-month survival curves for various endpoints between the 2 subgroups (BiOSS and rDES patients). If a particular endpoint occurred for a given patient more than once in a 48-month follow-up period, then survival time was treated as the time until the first occurrence of this event. Notably, when considering MACEs (which represent a composite endpoint), the survival time was defined as the period leading up to the occurrence of the first event among the following: all-cause death, cardiac death, MI, or TLR.

Univariable and multivariable Cox regression analyses (utilizing the Cox proportional hazards model) were conducted to assess differences in survival rates among the groups. The multivariable Cox regression model was selected through stepwise selection, employing a backward elimination algorithm with a significance threshold set at 0.1. The outcomes, including the hazard ratio (HR) and the corresponding 95% confidence intervals for the HR, were subsequently reported.

Statistical analyses were performed using GraphPad Prism 10 for mac OS software (Sonoma 14.4.1).

## 3. Results

### 3.1. Baseline Vharacteristics

**Figure 2:**
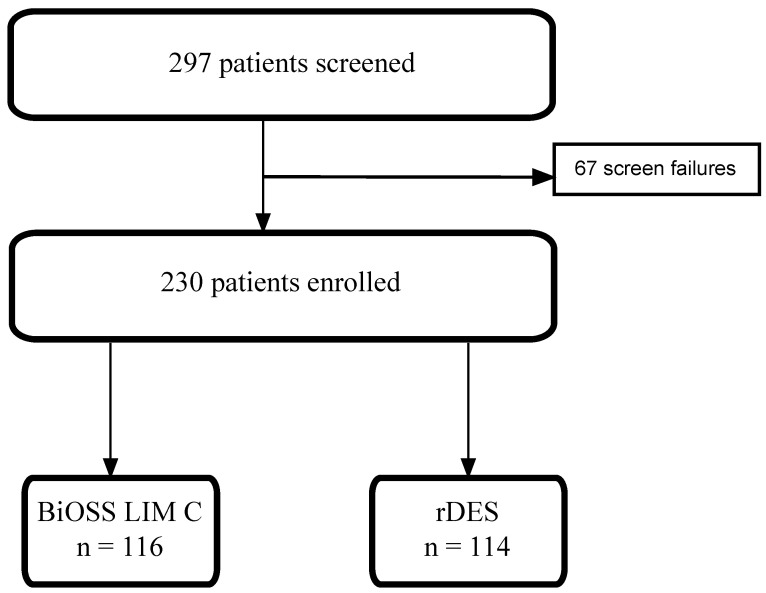
Study flow chart. rDES—regular drug-eluting stent.

Out of 297 screened patients, we enrolled 230 patients, sorting 116 patients into the BiOSS LIM C group and 114 patients into the rDES group (Figure 2). The mean ages of the patients in the BiOSS and rDES groups were 62.7 ± 10.6 and 65.8 ± 8.4 years old (*p* = 0.01), respectively. Patients in the rDES group had higher rates of prior MI (47.4% vs. 31.9%, *p* = 0.02) and prior CABG (10.5% vs. 2.6%, *p* = 0.02). Most procedures were performed as elective PCIs (BiOSS vs. rDES: 48.3% vs. 59.6%, *p* = 0.09) and were performed in bifurcations within the LAD/diagonal branch (BiOSS vs. rDES: 51.7% vs. 61.4%, *p* = 0.15). Numerically, true bifurcations were more frequent in the BiOSS group (66.4% vs. 56.1%, *p* = 0.13) (Table 2).

### 3.2. Procedure Characteristics

The successful device implantation rate was 100% in both groups. MV predilating was performed in almost all cases (BiOSS vs. rDES: 98.3% vs. 94.7%, *p* = 0.17), and SB predilating was performed in less than 40% in both groups (BiOSS vs. rDES: 37.9% vs. 38.6%, *p* = 0.99). Stents in the BiOSS group had larger proximal diameters (3.36 ± 0.32 mm vs. 3.07 ± 0.40 mm, *p* < 0.01) and were shorter (19.5 ± 3.6 mm vs. 22.1 ± 6.3 mm, *p* < 0.01). The use of the proximal optimization technique was much lower in the BiOSS group (20.7% vs. 47.4%, *p* < 0.01). Stents implanted in SBs were comparable (BiOSS vs. rDES: 21.6% vs. 17.5%, *p* = 0.62) (Table 3).

### 3.3. Clinical Outcomes

**Figure 3:**
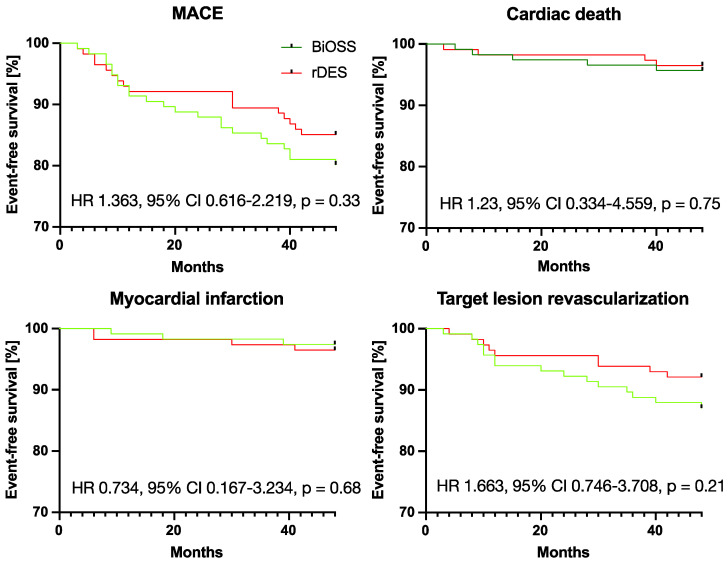
Kaplan–Meier curves showing 48-month outcomes. MACE—major adverse cardiovascular event.

We obtained data from all patients in both groups. Detailed data on MACE, death, cardiac death, MI, and TLR rates are provided in Table 4 and Figure 3. At 48 months, there were no statistically significant differences between the BiOSS and rDES groups in terms of MACE, cardiac death, MI, or TLR. There were no stent thrombosis cases in any of the groups. In detail, the results were as follows for BiOSS vs. rDES: MACEs—18.1% vs. 14.9%, HR 1.36, 95% CI 0.62–2.22, and *p* = 0.33; cardiac death—4.3% vs. 3.5%, HR 1.23, 95% CI 0.33–4.56, and *p* = 0.75, MI (2.6% vs. 3.5%, HR 0.73, 95% CI 0.17–3.23, and *p* = 0.68; and TLR—11.2% vs. 7.9%, HR 1.66, 95% CI 0.75–3.71, and *p* = 0.21. All MACE cases were reported as serious adverse events.

### 3.4. Predictors of MACE and TLR

To identify the potential predictors of MACE and TLR in the study population, we initially performed a univariable analysis (Supplementary Tables S1 and S2); here, we present only multivariable analyses for MACE (Table 5) and TLR (Table 6) for the BiOSS and rDES subgroups.

In the case of the BiOSS group, the MACE rate was lower in women (HR 0.43). PCI in NSTEMI/UA (HR 1.60), PCI within true bifurcations (HR 1.34), MV predilatation (HR 1.89), and prior MI (HR 2.21) were predictors associated with higher MACE rates. In the rDES group, diabetes (HR 1.98) and the POT (HR 0.23) were identified as additional important predicting factors.

In the case of the BIOSS group, PCI within true bifurcations (HR 3.78), MV predilatation (HR 1.09), and true bifurcations (HR 1.23) were predictors associated with higher TLR rates. In the rDES group, diabetes (HR 2.33) and the POT (HR 0.23) were identified as additional important predicting factors.

## 4. Discussion

Here, we present the 4-year results of a randomized controlled study in which coronary bifurcations were treated. The success rate of the implantation of the BiOSS LIM C stent was very high. The cumulative rates of MACEs were comparable between the BiOSS LIM C and rDES groups. Therefore, the POLBOS 3 trial sets an important benchmark for treating non-LM coronary bifurcations. Nevertheless, because of an inadequate sample size (premature study termination), it was not possible to confirm or refute the non-inferiority of BiOSS to rDES stents.

Gasior et al. analyzed overexpansion capabilities and thrombogenicity at the SB ostium after stent implantation in an in vitro bifurcation model. The authors used four DES: BiOSS LIM C and three rDESs (Xience Sierra, Ultimaster, and Biomime) [12]. Interestingly, when conducting confocal microscopy, the thrombus area was the smallest in the BiOSS LIM C and statistically significant when compared with Biomime stents (Bioss LIM C vs. Biomime: 0.21 mm^2^ vs. 4.80 mm^2^, *p* < 0.01). Also, areas with a high shear rate (>1000 s^−1^) and the maximum shear rate were numerically an in vivo study (pigs), in which BiOSS LIM C and Orsiro stents yielded similar results [11]. After 28 days, optical coherence tomography (OCT) verified the patency of all stents without any evidence of thrombus formation. A morphometric analysis revealed no discernible variances between the groups in terms of stent diameter (*p* = 0.141), neointima area (*p* = 0.247), percentage of area stenosis (*p* = 0.293), or percentage of diameter stenosis (*p* = 0.069). Additionally, no notable variations were observed between the groups in terms of their histopathology scores. Across all groups, injury and inflammation scores remained low (mean grade < 1).

In comparison with previous studies on the BiOSS stent (POLBOS 1 and POLBOS 2), the results were better. In the current study, the results were as follows for BiOSS vs. the rDESs: MACEs (18.1% vs. 14.9%, *p* = 0.33), cardiac death (4.3% vs. 3.5%, *p* = 0.75), MI (2.6% vs. 3.5%, *p* = 0.68), and TLR (11.2% vs. 7.9%, *p* = 0.21). The 4-year clinical outcomes following POLBOS 1 and POLBOS 2 were a bit worse. At 48 months, the MACE rate was 19.8% in the BiOSS group and 18.8% in the rDES group (*p* = 0.64), whereas the TLR rates were 15.3% and 12.1%, respectively (*p* = 0.34) [18].

The results of POLBOS 3 are quite comparable to those observed in the first-in-human study with the BiOSS LIM C [13]. Similarly, the device success rate was 100%. However, the rDES was deployed in the SB at a lower incidence. At 12 months, the MACE rate was 9.5% (our current study: 8.6%), and clinically driven TLR was 6.3% (our current study: 6.0%). It is worth stressing that in the current study, the rate of POT was lower (20.7% vs. 53.7%). This is against current recommendations; however, it might be associated with the BiOSS structure [4,19]. Also, POT rates below 40% can be observed in other studies [20,21,22,23,24].

In the EPIC3-BIOSS prospective study, 124 patients in whom the BiOSS LIM C stent was deployed were enrolled [25]. The stent was successfully deployed in 97.6% of patients, and in 14.5% of cases, a double-stenting technique was used. True bifurcations were present in 47.2% of cases, and the POT was performed in 26.6% of cases. At 12 months, the MI rate was 2.72%, the TLR rate was 5.4%, and the stent thrombosis rate was 0.9%. The rates were comparable to those in our study, and in our study, we included more true bifurcation cases.

A new registry with BiOSS LIM C was recently published—IBIOSS [26]. Interestingly, only patients with true bifurcations were included. Although 207 patients were enrolled, 1.1.1. bifurcations comprised only 30.9% (in our paper: 62.9%). The double-stenting technique was used in 9.3% of cases, and the POT was used in 11.7% of cases. At a mean follow-up of 24.1 ± 19.5 months, the MACE rate was 11.1% (in our study: 12.2%), the TLR rate was 2.9%, and the MI rate was 2.9%.

The data obtained in POLBOS 3 are similar to those observed in the literature. Among the entire cohort of patients in the LEADERS trial, those who underwent percutaneous coronary interventions for bifurcation lesions exhibited the following 5-year outcomes: a MACE rate of 35.3%, a cardiac death rate of 8.5%, an MI rate of 11.9%, and a TLR rate of 14.9% [27]. Furthermore, upon examining data from other dedicated bifurcation stents, the long-term data (over 3 years) of the Axxess stent revealed a MACE rate of 19.5%, a cardiac death rate of 2.0%, an MI rate of 7.4%, and a clinically driven TLR rate of 10.1% [28]. Findings from a separate cohort demonstrated even more favorable outcomes for the Axxess stent at 5 years [29]. At 2 years, the extended data for Tryton revealed a MACE rate of 23.8%, a cardiac death rate of 4.4%, an MI rate of 10.2%, and a TLR rate of 9.2% [30]. For Stentys, at 5 years, the incidence of target vessel failure was 22.8%, with a notably high rate of stent thrombosis at 4.5%. [31]. Also, Riku et al. recently showed that late TLR cases continued over 10 years at a rate of 2.4%/year in a complex PCI group and 1.1%/year in a noncomplex PCI group [32].

In our study, we identified the following factors predicting of MACEs and TLR. In the case of the BiOSS group, the MACE rate was lower in women (HR 0.43). PCIs in NSTEMI/UA (HR 1.60), PCIs within true bifurcations (HR 1.34), MV predilatation (HR 1.89), and prior MI (HR 2.21) were predictors associated with higher MACE rates. In the rDES group, diabetes (HR 1.98) and the use of the POT (HR 0.23) were identified as additional important predicting factors. In the case of the BIOSS group, PCIs within true bifurcations (HR 3.78), MV predilatation (HR 1.09), and true bifurcations (HR 1.23) were predictors associated with higher TLR rates. In the rDES group, diabetes (HR 2.33) and the POT (HR 0.23) were identified as additional important predicting factors. It is worth stressing that the POT was a predicting factor in the rDES group and not in the BiOSS group. This may be associated with the BiOSS stent’s structure. They are in agreement with other studies. Within the Milan and New Tokyo (MITO) Registry, calcification, true bifurcation, and insulin-dependent diabetes were identified as independent predictors of in-stent restenosis within MB [33]. In another research study, the primary factors influencing MACE rates were identified as the utilization of a two-stent technique and the presence of diabetes [34]. Chen et al. similarly verified the correlation between two-stent deployment and increased MACE rates. The researchers proposed that among the various two-stent techniques, the double-kissing (DK) crush yielded the most advantageous results [35].

In the end, it is rightful to raise the issue of using provisional T-stenting vs. two-stent techniques. Fujisaki et al. conducted a meta-analysis comprising 13 randomized controlled trials (RCTs) that compared provisional with dedicated bifurcation stent placements employing various PCI techniques [36]. A notable aspect of their study is the incorporation of RCTs primarily or exclusively utilizing second-generation drug-eluting stents. Previous meta-analyses on bifurcation PCIs encompassed studies employing bare metal stents or first-generation drug-eluting stents which are now considered outdated. We concur that utilizing contemporary data is preferable for gaining insights into future approaches in this field. This meta-analysis reinforced several key findings. Firstly, advancements in newer-generation stents, alongside improved guidewires, support catheters, adjunctive therapies, and imaging modalities, have resulted in enhanced two-stent strategies for managing coronary bifurcation lesions [3]. Secondly, among complex lesion subsets, the DK crush technique emerges as superior to alternative approaches [6,37]. Lastly, while a dedicated two-stent strategy appears more effective than a provisional strategy in treating complex bifurcations, the superiority of a two-stent strategy in “noncomplex” bifurcations remains uncertain [38,39]. Also, smaller bifurcations (non-LM bifurcations), as our study shows, can be safely treated with a provisional stenting technique. Interestingly, Jones et al. showed promising results with provisional bifurcation stenting and sirolimus-eluting balloon use in the SB [40].

### Study Limitations

The POLBOS 3 study has several limitations that should be acknowledged. First, the study was prematurely terminated, and the initial sample size calculations were not applicable. Using various stent types and drugs within the control group presents a limitation, although this approach aimed to mimic real-world clinical scenarios. The predetermined randomization scheme might have influenced investigators’ decisions to enroll patients based on specific angiographic characteristics. Moreover, disparities in final kissing balloon/POT strategies between the two study groups (randomization in the rDES group versus operator discretion in the BiOSS group) could have impacted the outcomes as well.

## 5. Conclusions

The implantation success rate of the BiOSS LIM C stent was very high, and cumulative rates of MACEs were comparable between the BiOSS LIM C and rDES groups, although the study population was underpowered due to premature study termination. These results show the function of dedicated bifurcation stents, especially when a proper deployment protocol is applied.

## Figures and Tables

**Table 1:** Inclusion and exclusion criteria.

Inclusion Criteria	Exclusion Criteria
The subject is at least 18 years of age. The subject is able to verbally confirm their understanding of the risks and benefits of receiving PCI for bifurcation lesions, and they or their legally authorized representative provide written informed consent prior to any study-related procedure. The target main branch lesion(s) is located in a native coronary artery and has a diameter measuring ≥2.5 mm and ≤4.5 mm. The target side branch lesion(s) located in a native coronary artery with a diameter of ≥2.0 mm. The target lesion(s) is amenable for PCI with balloon angioplasty of the side branch.	Non-cardiac comorbidities are present with a life expectancy < 1 year or may result in protocol non-compliance (per the site investigator’s medical judgment). Subjects who refuse to give informed consent. Subjects with an LVEF < 30%. Subjects with a moderate or severe degree of valvular heart disease or primary cardiomyopathy. Patients with distal left main stenosis. Bifurcation lesions requiring the two-stent technique a priori.

PCI—percutaneous coronary intervention; LVEF—left ventricular ejection fraction.

**Table 2:** Baseline characteristics.

Parameter		Total Population N = 230 (%)	BiOSS Group n = 116 (%)	rDES Group n = 114 (%)
Age [years]		64.3 ± 9.6	62.7 ± 10.6	65.8 ± 8.4 *
Women		45 (19.6)	22 (19)	23 (20.2)
Hypertension		183 (79.6)	97 (83.6)	86 (75.4)
Hypercholesterolemia		230 (100)	116 (100)	114 (100)
Diabetes type 2		80 (34.8)	38 (32.8)	42 (36.8)
Prior myocardial infarction		91 (39.6)	37 (31.9)	54 (47.4) *
Prior PCI		114 (49.6)	50 (43.1)	64 (56.1)
Coronary artery bypass grafting		15 (6.5)	3 (2.6)	12 (10.5) *
Peripheral artery disease		15 (6.5)	5 (4.3)	10 (8.8)
Chronic kidney disease		28 (12.2)	10 (8.6)	18 (15.8)
History of smoking		79 (34.3)	35 (30.2)	44 (38.6)
Clinical indication for PCI				
	Planned PCI	124 (53.9)	56 (48.3)	68 (59.6)
	Unstable angina	34 (14.8)	22 (19)	12 (10.5)
	NSTEMI	41 (17.8)	29 (25)	12 (10.5)
	STEMI	31 (13.5)	9 (7.8)	22 (19.4)
Lesion location				
	LAD	130 (56.5)	60 (51.7)	70 (61.4)
	LCx	58 (25.2)	22 (18.9)	36 (31.6)
	RCA	42 (18.3)	34 (29.3)	8 (7.0)
SYNTAX score		22.8 ± 3.7	23.1 ± 3.9	22.5 ± 4.1
Medina classification				
	1.1.1.	131 (56.9)	73 (62.9)	58 (50.9)
	1.0.1.	6 (2.6)	4 (3.4)	2 (1.8)
	0.1.1.	4 (1.7)	0	4 (3.5)
	1.0.0.	12 (5.2)	0	12 (10.5)
	0.1.0.	6 (2.6)	6 (5.2)	0
	1.1.0.	71 (30.9)	33 (28.4)	38 (33.3)
True bifurcation	1.1.1./1.0.1./0.1.1.	141 (61.3)	77 (66.4)	64 (56.1)

* *p* < 0.05; LAD—left anterior descending artery; LCx—left circumflex artery; NSTEMI—non-ST-elevation myocardial infarction; PCI—percutaneous coronary intervention; RCA—right coronary artery; rDES—regular drug-eluting stent; STEMI—ST-elevation myocardial infarction.

**Table 3:** Procedural characteristics.

Parameter	Total Population N = 230 (%)	BiOSS Group n = 116 (%)	rDES Group n = 114 (%)
Successful implantation	230 (100)	116 (100)	114 (100)
Main vessel predilatation	222 (96.5)	114 (98.3)	108 (94.7)
Side branch predilatation	88 (38.3)	44 (37.9)	44 (38.6)
Predilation of both branches	34 (14.8)	8 (6.9)	26 (22.8) *
Nominal stent diameter in main vessel [mm]	3.22 ± 0.34	3.36 ± 0.32	3.07 ± 0.40 *
Nominal stent diameter in main branch [mm]	-	2.73 ± 0.31	-
Nominal stent length [mm]	20.8 ± 4.8	19.5 ± 3.6	22.1 ± 6.3 *
Side branch postdilatation	52 (22.6)	28 (24.1)	24 (21.1)
Proximal optimization technique	78 (33.9)	24 (20.7)	54 (47.4) *
Final kissing balloon technique	60 (26.1)	28 (24.1)	32 (28.1)
Stent in a side branch	45 (19.6)	25 (21.6)	20 (17.5)
Dissection requiring an additional stent in a main vessel—main branch	5 (2.2)	3 (2.6)	2 (1.8)
Fluoroscopy time [min]	12.8 ± 5.9	13.14 ± 5.6	12.5 ± 7.3
Contrast volume [mL]	196.8 ± 70.2	203.8 ± 78.7	189.6 ± 71.9
Vascular access femoral/radial	0/100%	0/100%	0/100%
Guiding catheter 6F/7F	100%/0	100%/0	100%/0

* *p* < 0.05; rDES—regular drug-eluting stent.

**Table 4:** Forty-eight-month outcomes.

Outcome	1 Year	2 Years	3 Years	4 Years
BiOSS LIM C				
MACE	10 (8.6)	14 (12.1)	18 (15.5)	21 (18.1)
Death	3 (2.6)	4 (3.4)	6 (5.2)	8 (6.7)
Cardiac death	2 (1.7)	3 (2.6)	4 (3.4)	5 (4.3)
MI	1 (0.9)	2 (1.7)	2 (1.7)	3 (2.6)
TLR	7 (6.0)	9 (7.8)	12 (10.3)	13 (11.2)
rDES				
MACE	9 (7.9)	9 (7.9)	12 (10.5)	17 (14.9)
Death	4 (3.5)	4 (3.5)	5 (4.4)	6 (5.2)
Cardiac death	2 (1.8)	2 (1.8)	2 (1.8)	4 (3.5)
MI	2 (1.8)	2 (1.8)	3 (2.6)	4 (3.5)
TLR	5 (4.4)	5 (4.4)	7 (6.1)	9 (7.9)

rDES—regular drug-eluting stent; MACE—major adverse cardiovascular event; MI—myocardial infarction; TLR—target lesion revascularization.

**Table 5:** Multivariable logistic regression for MACE.

Parameter	Multivariable Analysis	
	HR (95% CI)	*p*-Value
BIOSS		
Female vs. male	0.43 (0.29–0.88)	0.02
NSTEMI/UA	1.60 (1.13–3.99)	0.03
Prior myocardial infarction	2.21 (1.44–4.67)	0.02
True bifurcation	1.34 (1.09–3.03)	0.01
Main vessel predilatation	1.89 (1.56–4.04)	0.02
rDES		
Female vs. male	0.31 (0.19–0.44)	0.01
NSTEMI/UA	2.13 (1.44–3.09)	0.04
Prior myocardial infarction	2.53 (1.89–7.04)	0.02
True bifurcation	2.44 (1.09–5.90)	<0.01
Diabetes	1.98 (1.34–5.12)	0.02
Proximal optimization technique	0.23 (0.14–0.43)	<0.01

MACE—major cardiovascular adverse event; NSTEMI—non-ST-elevation myocardial infarction; UA—unstable angina; CI—confidence interval; HR—hazard ratio.

**Table 6:** Multivariable logistic regression for TLR.

Parameter	Multivariable Analysis	
	HR (95% CI)	*p*-Value
BiOSS		
Prior myocardial infarction	3.78 (2.43–11.01)	<0.01
True bifurcation	1.23 (1.13–3.23)	0.01
Main vessel predilatation	1.09 (1.02–2.99)	0.04
rDES		
Prior myocardial infarction	4.22 (1.78–10.09)	<0.01
True bifurcation	2.87 (1.67–7.02)	<0.01
Diabetes	2.33 (1.56–5.33)	0.02
Proximal optimization technique	0.23 (0.05–0.49)	0.01

CI—confidence interval; HR—hazard ratio; TLR—target lesion revascularization.

## Data Availability

Data are available from the corresponding author upon request.

## Supplementary Materials

The following supporting information can be downloaded at https://www.mdpi.com/article/10.3390/biomedicines12050938/s1, Table S1: Univariable Cox regression for MACEs; Table S2: Univariable Cox regression for TLR.
