# Supplementary material for: The Randomized, Multicenter, Open-Label, Controlled POLBOS 3 Trial Comparing Regular Drug-Eluting Stents and the Sirolimus-Eluting BiOSS LIM C Dedicated Coronary Bifurcation Stent: Four-Year Results
Source: Biomedicines. 2024 Apr 23;12(5):938. doi: 10.3390/biomedicines12050938 (PMC11118130; doi:10.3390/biomedicines12050938)
Supplement: Supplementary file 1 [file biomedicines-12-00938-s001.zip › biomedicines-2971077-supplementary.pdf]

Supplementary Table S1. Univariable regression for MACE

Parameter	Univariable analysis	
	HR (95% CI)	p-value
BiOSS vs. DES	1.018 (0.407 – 1.777)	0.647
Female vs. male	0.547 (0.360 – 0.993)	0.049*
NSTEMI/UA	2.680 (1.340 – 4.986)	0.006*
Diabetes type 2	1.754 (0.941 – 2.015)	0.458
Prior MI	2.501 (1.349 – 5.346)	0.019*
True Bifurcation	2.779 (1.114 – 4.751)	0.011*
Prior CABG	1.231 (0.893 – 2.318)	0.671
Main vessel predilatation	2.020 (1.548 – 3.922)	0.039*
Final kissing balloon	0.601 (0.396 – 1.650)	0.326
Proximal optimization technique	0.201 (0.108 – 0.552)	0.001*
Side branch stenting	1.452 (0.645 – 2.441)	0.541

DES, drug-eluting stent; *MACE*, major cardiovascular adverse events; *NSTEMI*, non-ST elevation myocardial infarction; *UA*, unstable angina
*statistically significant

Supplementary Table S2. Univariable logistic regression for TLR

Parameter	Univariable analysis	
	HR (95% CI)	p-value
BiOSS vs. DES	1.280 (0.630 – 2.455)	0.325
Diabetes type 2	1.635 (0.887 – 2.940)	0.183
Prior MI	3.221 (1.736 – 9.384)	0.002*
True bifurcation	2.980 (1.003 – 9.672)	0.023*
Prior CABG	1.432 (0.715 – 2.113)	0.532
Main vessel predilatation	2.120 (1.338 – 3.322)	0.039*
Final kissing balloon	0.527 (0.287 – 1.018)	0.081
Proximal optimization technique	0.291 (0.176 – 0.431)	<0.001*
Side branch stenting	1.689 (0.735 – 3.124)	0.321

CI, confidence interval; *DES*, drug-eluting stent; *OR*, odds ratio; *TLR*, target lesion revascularization;
*statistically significant
